# Comment on: Population pharmacokinetics and dosing optimization of mezlocillin in neonates and young infants

**DOI:** 10.1093/jac/dkac305

**Published:** 2022-09-14

**Authors:** Joseph F Standing

**Affiliations:** Infection, Immunity and Inflammation, Great Ormond Street Institute of Child Health, University College London, London, UK; Pharmacy Department, Great Ormond Street Hospital for Children NHS Foundation Trust, London, UK

Zhou and colleagues^1^ have studied the pharmacokinetics of mezlocillin in neonates and used the resulting model to simulate probability of target attainment (PTA) in order to inform dosing. This is an important study on an agent with a paucity of neonatal dosing information. To interpret the model correctly however, its mathematical properties need to be fully understood.

Recently, authors in this Journal have found the scaling of clearance in neonates challenging.^2^ Allometric size scaling with a coefficient of 0.75 does not work well in children <2 years of age^3^ and in addition to postmenstrual age (PMA), the first week of life sees rapid changes in clearance and the washout of maternal creatinine.

Previous neonatal pharmacokinetic studies published in this Journal have biologically informed fixed covariates based on expected size and PMA effects,^4,5^ and when patients in the first week of life (regardless of gestational age) are studied, a further postnatal age effect has been estimated.^6^ Maternal creatinine washout with subsequent age-related rises in creatinine have also been accounted for, and doing so improves predictive performance over other approaches when compared by fitting an external, prospectively collected, multi-centre dataset.^7^

Zhou and colleagues^1^ have taken a different approach however, estimating an empirical PMA effect with a power model and adding an oddly parameterized exponential term as follows:

$$CL=\theta_{4}{\left(\frac{CW}{2300}\right)}^{0.75}F_{age}\times RF$$

where *θ*4 was estimated to be 0.18 L/h representing the typical value of clearance for a neonate weighing 2300 g, PMA of 35.8571 weeks and serum creatinine (CREA) of 73 μmol/L, *F_age_* was (PMA/35.8571)^0.212^ and RF defined as EXP[(CREA − 73) × 0.155].

As written above and reported in Table 2 of the paper,^1^ this cannot possibly have been the model fitted to the data. Zhou *et al*. report a serum creatinine range of 25–209 μmol/L. Substituting 25 into the equation for RF above gives a value of 0.00058 and reduces clearance by almost 1000-fold, whereas substituting 209 into the equation yields a value of 1.43 × 10^9^. Since RF multiplies clearance and even much smaller deviations in creatinine give physiologically implausible clearance values there is clearly an error in parameter reporting, the second such error in consecutive peer-reviewed neonatal PK studies in this Journal.^2^

Whilst ‘all covariate models are wrong’ and of course authors are free to choose how to parameterise weight, age and creatinine effects in neonatal antimicrobial pharmacokinetic models, when everyone choses different empirically derived methods, not only does it make interpreting model parameters and comparing values across studies difficult, but it also increases the chance of reporting errors such as this. Mechanistic weight, age maturation, and maternal creatinine-corrected models are available in the literature, including in previously published studies in this Journal.^4^
